# The Bradshaw Lecture

**Published:** 1896-12-19

**Authors:** 


### THE BRADSHAW LECTURE.

The remarks made by Mr. Reginald Harrison in the Bradshaw Lecture on Vesical, Stone, and Prostatic Disorders, in regard to the advantages of perineal lithotomy and lithotrity are worthy of careful attention in these days, when the surgical tendency drifts so decidedly towards the suprapubic method. When vesical calculus occurs in elderly men the prostate often requires as much consideration as the stone in deciding on the course of treatment to be adopted, and where the prostate is not only enlarged but has been more or less disorganised by repeated attacks of inflammation, a mere removal of the stone, as, for example, by the suprapubic method, by no means places the patient in the best position for recovery. Mr. Harrison points out the advantage of perineal drainage, and strongly recommends, for the cases in which, by reason of the condition of the bladder and urinary apparatus generally, the ordinary crushing operation is not applicable, the performance of perineal lithotrity. An ordinary boutonière, or median perineal cystotomy, is practised on a grooved staff sufficient to admit the introduction of the finger, and the stone is caught by crushing forceps.

